# Treatment for non-tuberculous mycobacteria: challenges and prospects

**DOI:** 10.3389/fmicb.2024.1394220

**Published:** 2024-06-03

**Authors:** Liberty E. Conyers, Bernadette M. Saunders

**Affiliations:** School of Life Sciences, University of Technology Sydney, Sydney, NSW, Australia

**Keywords:** Non-tuberculous mycobacteria, *Mycobacterium avium*, *Mycobacterium abscessus*, clinical trials, antibiotics, treatment regime

## Abstract

Non-Tuberculous mycobacteria (NTM) are opportunistic environmental bacteria. Globally, NTM incidence is increasing and modeling suggests that, without new interventions, numbers will continue to rise. Effective treatments for NTM infections remain suboptimal. Standard therapy for *Mycobacterium avium* complex, the most commonly isolated NTM, requires a 3-drug regime taken for approximately 18 months, with rates of culture conversion reported between 45 and 70%, and high rates of relapse or reinfection at up to 60%. New therapeutic options for NTM treatment are urgently required. A survey of ongoing clinical trials for new NTM therapy listed on ClinicalTrials.Gov using the terms ‘*Mycobacterium avium*’, ‘*Mycobacterium abscessus*’, ‘*Mycobacterium intracellulare*’, ‘Non tuberculous Mycobacteria’ and ‘Nontuberculous Mycobacteria’ and a selection criterion of interventional studies using antibiotics demonstrates that most trials involve dose and combination therapy of the guideline based therapy or including one or more of; Amikacin, Clofazimine, Azithromycin and the anti-TB drugs Bedaquiline and Linezolid. The propensity of NTMs to form biofilms, their unique cell wall and expression of both acquired and intrinsic resistance, are all hampering the development of new anti-NTM therapy. Increased investment in developing targeted treatments, specifically for NTM infections is urgently required.

## 1 Introduction

Non-Tuberculous Mycobacteria (NTM) are environmental bacteria found commonly in soil and water, both natural and municipal sources. This group includes all members of the mycobacteria family excluding *Mycobacterium tuberculosis complex (TB)* and *Mycobacterium leprae*, the causative agents of tuberculosis and leprosy, respectively. NTMs represent a vast group of bacteria with over 190 distinct species (Armstrong et al., 2023). The most common causing disease in humans are the *Mycobacterium avium* complex (MAC), comprised of *Mycobacterium avium* and *Mycobacterium intracellulare,* then *Mycobacterium abscessus* (MAB) (Falkinham, 2013a). Obtaining accurate data on the extent of NTM infection is challenging, as NTMs are not notifiable diseases in many states or countries. Data from Queensland, Australia where NTMs are a notifiable disease, demonstrates that the number of NTM cases more than doubled in the last 10 years from 672 in 2012 (Thomson et al., 2017) to 1,490 cases in 2022 (Queensland Health, 2024). Current projections suggest NTM infections will triple by 2040 (Ratnatunga et al., 2020). Isolation of NTM are now eight times more common in Queensland than *M. tuberculosis*, with 191 cases of TB reported in 2023 versus 1,565 NTM isolated (Queensland Health, 2024). While isolation of *M. tuberculosis* indicates clinical disease, the clinical relevance of an NTM isolation and the decision to treat is more contentious. Clinical, microbiological, and radiological guidelines developed by the ATS/IDSA are used to clarify the likelihood of NTM pulmonary disease versus colonization (Griffith et al., 2007; Schiff et al., 2019).

Increases in NTM infections have also been identified on multiple continents including North America, Europe, and Asia (Andréjak et al., 2010; Lai et al., 2010; Moore et al., 2010; Park et al., 2010; Taiwo and Glassroth, 2010; Donohue and Wymer, 2016; Diel et al., 2017a). South Korea reported a 5-fold increase in NTM infections between 2007 and 2016 from 6.7 cases per 100,000 to 39.6 per 100,000 with MAC and *M. abscessus* being the most frequently reported (Lee et al., 2019). Japan has also seen a rapid increase in NTM cases 5.7 cases per 100,000 in 2007 to 14.7 cases per 100,000 by 2014, with MAC again, being the most prevalent NTM identified (Namkoong et al., 2016). This incidence rate was higher than the rate of TB in Japan in 2014 at 12.9 cases per 100,000 population (Namkoong et al., 2016).

Multiple theories have been posed to explain the increased numbers of NTMs reported in recent years. Improvements in diagnostic techniques is making accurate diagnosis easier, with faster and more specific tests able to identify NTM infections. The number of individuals who are immunocompromised, and therefore vulnerable to NTM infection is on the rise. A NHIS survey reported an increase from 2.7% in 2013 to 6.6% in 2021 in immunocompromised individuals (Martinson and Lapham, 2024). Individuals with cystic fibrosis (*CF*), who thankfully show an increase in life expectancy are unfortunately, also at increased risk of developing an NTM infection (Ruseckaite et al., 2022).

The changing climate may be another reason for infection rates rising. A retrospective study by Sherrard et al. compared NTM infection rates between those living in tropical and subtropical regions of Queensland (Sherrard et al., 2017). Those living in tropical regions were 2.5 times more likely to be impacted by an NTM infection (Sherrard et al., 2017). Similarly, a large geographic study conducted across the United States found areas with a higher risk of NTM infection had greater levels of precipitation, evapotranspiration and higher average temperatures (Adjemian et al., 2012). In the tropical state of Hawaii, NTM cases *per capita* were four times higher than mainland states (Adjemian et al., 2012) with five novel mycobacterial species recently identified in Hawaiian soils (Hennessee et al., 2009). It is also possible that the tropical climate leads to more outdoor activities, such as hiking and swimming which increase chances of exposure to these bacteria (Koschel et al., 2006). With the changing climate, and expansion of tropical regions, this may increase the risk of NTM infection.

Even with increasing instances of infection, NTMs also have the significant issue of misdiagnosis. Clinically, NTM infections manifest with non-specific symptoms such as chronic cough, weight loss, fatigue and fever (Griffith et al., 2007). Diagnosis requires both clinical and microbiologic confirmation, including pulmonary symptoms, nodular or cavity opacities observed through imaging and positive cultures from sputum, bronchial wash or lavage or lung biopsy with mycobacterial histopathologic features (Griffith et al., 2007). NTMs can be misdiagnosed as TB, especially in areas where TB is endemic (Baldwin et al., 2019). One final and important distinction is that a positive NTM isolation does not always signify infection or disease. This further complicates the choice to begin treatment, or continue monitoring the patient, individual patient risk factors then need to be considered.

The general population is frequently exposed to *M. avium* and other NTMs during everyday activities. A common source of exposure occurs from showerheads, as bacteria found in the municipal water are aerosolized and inhaled (Gebert et al., 2018). NTMs are also found in soils when gardening and can be isolated from hospital equipment (Falkinham, 2013b). Despite the population being frequently exposed to NTM, only a very small percentage develop infection, with immunocompromised individuals at increased risk. NTM infection has been identified in 3.7–24% of *CF* patients with MAC and MAB being the most prevalent (Olivier et al., 2003; Levy et al., 2008; Roux et al., 2009; Hill et al., 2012). NTM infection in *CF* patients results in declined lung functionality and overall reduced life expectancy (Malouf and Glanville, 1999). HIV positive patients are also susceptible to NTM infections (Chai et al., 2022). Bacteria can disseminate from the lungs to anywhere in the body, with bone marrow, liver, spleen and lymph nodes the most common (Horsburgh, 1991; von Reyn et al., 2002). Inexplicably, another group with increased susceptibility to NTM infection, are slender, tall immunocompetent women between the ages of 50–80 (Chan and Iseman, 2010; Takayama et al., 2022). Often referred to as ‘Lady Windemere’ syndrome, studies have shown that women meeting these criteria are more likely to develop NTM infections. While chronic cough suppression has been hypothesized as a risk factor for their developing MAC disease no direct evidence explaining this increased susceptibility has been established. Some theories include decreased estrogen in post-menopausal women, or abnormalities in fibrillin which result in expression of immunosuppressive TGF-β may lead to greater susceptibility to NTM infections (Chan and Iseman, 2010).

These significant global increases make having an effective treatment for NTM disease increasingly important. Current treatment regimens are not optimal for efficient and sustained clearance of NTM infection. More research to elucidate antibiotics which are effective against NTM is essential. This review aims to highlight the significant challenges related to treatment of NTM infection, with compounding factors such as limited clinical trials, the paucity of NTM drug discovery pipelines and intrinsic and adaptive drug resistance of the pathogen all contributing to this challenge. It will also discuss opportunities of using new therapeutics to tackle this significant and growing problem.

## 2 Current treatment regime

### 2.1 Challenges of current treatment options

The current treatment for NTM infection is a minimum of three antibiotics. Most regimes include ethambutol and rifampicin with a macrolide backbone of either clarithromycin or azithromycin(Griffith et al., 2007). Due to the genetic variability of NTMs strains there is not one standardised treatment plan. Treatment is continued 12 months post a negative sputum culture(Griffith et al., 2007). However, studies report between 10 and 60% of patients experience relapse or reinfection within 6–12 months of initial therapy completion (Lee et al., 2015; Koh et al., 2017; Kwon et al., 2019). One possible reason for the poor outcome is the pharmacokinetic interactions between antibiotics. A study by van Ingen et al. identified that rifampicin significantly reduced peak serum concentrations of macrolides, when used concurrently, clarithromycin and azithromycin concentrations were decreased by up to 68 and 23%, respectively (Van Ingen et al., 2012). If this decrease in serum concentration reduced macrolide killing efficacy in the lung is unknown. Macrolides have strong tissue penetration therefore it is possible therapeutic doses were still being achieved, however, the lack of synergy between these antibiotics may be reducing the efficacy of this treatment regime (Zuckerman et al., 2011).

### 2.2 Determination of the current treatment regime

The first “American Thoracic Society (ATS) declaration for Nontuberculous Mycobacteria identification and treatment” in 1990 recommended a four drug regimen including 300 mg isoniazid, 600 mg rifampin, and 25 mg/kg ethambutol for the first 2 months followed by 15 mg/kg for the remainder of treatment with streptomycin for the first three to 6 months of therapy (Wallace et al., 1990). This regimen was supported by data from two non-comparative clinical trials (Ahn et al., 1986; Seibert and Bass, 1989). In 1997, the regime was revised to incorporate the macrolides, clarithromycin or azithromycin, now a staple for MAC treatment (American Thoracic Society, 1997).

The ATS/IDSA recommendation for NTM treatment regime was based on data from a number of studies (Table 1) demonstrating that the inclusion of between 250 mg to 750 mg clarithromycin twice daily, to the ethambutol, rifampin and streptomycin regime was highly successful with conversion rates of between 78 and 92%, a low relapse rates of 18% and increased survival (Shafran et al., 1996; Wallace et al., 1996a). Subsequent studies investigating dosing frequency demonstrated a thrice weekly regimen was as effective while reducing toxicity to patients (Griffith et al., 1998, 2000, 2001). Recent studies have seen a shift from rifabutin to rifampin, as rifabutin was associated with higher rates of adverse events (Griffith et al., 2000, 2001). Rifabutin is still recommended as part of the ATS declaration, but is only used in more severe cases of MAC infection.

**Table 1:** Studies guiding the ATS/IDSA recommendations for treatment of *M. avium* disease.

Study Title	Year published	Antibiotic regime	Conversion rate	Reference
Clarithromycin Regimens for Pulmonary *Mycobacterium avium* Complex The First 50 Patients	1996	500 mg clarithromycin twice daily, ethambutol, rifampin or rifabutin and initial streptomycin	Culture conversion 92% (38/39 individuals who completed 5+ months of therapy) Relapse rate 18%	Wallace et al. (1996a)
A Comparison of Two Regimens for the Treatment of *Mycobacterium avium* Complex Bacteremia in AIDS: Rifabutin, Ethambutol, and Clarithromycin versus Rifampin, Ethambutol, Clofazimine, and Ciprofloxacin	1996	Regimen A Rifampin, ethambutol, clofazimine and ciprofloxacin Regimen B Rifabutin (daily), ethambutol (daily), and clarithromycin (twice daily)	Culture conversion Regimen A 29% Regimen B 69%	Shafran et al. (1996)
Initial (6-Month) Results of Three-Times-Weekly Azithromycin in Treatment Regimens for *Mycobacterium avium* Complex Lung Disease in Human Immunodeficiency Virus-Negative Patients	1998	Regimen A TIW azithromycin, daily ethambutol, daily rifabutin and initial twice weekly (BIW) streptomycin Regimen B TIW azithromycin, TIW ethambutol, TIW rifabutin and initial BIW streptomycin	Culture conversion Regimen A 74% Regimen B 62%	Griffith et al. (1998)
Early Results (at 6 Months) with Intermittent Clarithromycin-Including Regimens for Lung Disease Due to *Mycobacterium avium* Complex	2000	Regimens of clarithromycin, rifabutin, and ethambutol TIW	Culture conversion 78%	Griffith et al. (2000)
Azithromycin-Containing Regimens for Treatment of *Mycobacterium avium* Complex Lung Disease	2001	Group A Azithromycin 300 mg-600 mg/day with companion drugs Group B Azithromycin 600 mg TIW with companion drugs daily Group C Azithromycin 600 mg TIW and companion drug TIW Companion drugs rifabutin or rifampin, ethambutol and initial streptomycin	Culture conversion Group A 59% Group B 55% Group C 65%	Griffith et al. (2001)
The Effect of Combined Therapy According to the Guidelines for the Treatment of *Mycobacterium avium* Complex Pulmonary Disease	2003	Clarithromycin, ethambutol, rifampicin and initial streptomycin for 12 months	Culture conversion 57.7% Relapse rate 39%	Kobashi and Matsushima (2003)
A double-blind randomized study of aminoglycoside infusion with combined therapy for pulmonary *Mycobacterium avium* complex disease	2007	Regimen A Rifampicin, ethambutol, clarithromycin and streptomycin Regimen B Rifampicin, ethambutol and clarithromycin	Culture conversion Regimen A 71.1% Regimen B 47.2% Relapse rate Regime A 30.8% Regimen B 35.1%	Kobashi et al. (2007)

While the majority of studies demonstrate that the 3-drug regimen induces sputum conversion, the rate of initial conversion, and the rate of relapse, can vary significantly. Studies reported rates of sputum conversion between 47 and 92%, and a relapse rate of 18–39%, with a gradual decrease in rates of conversion evident over time (Table 1). This may be due growing rates of antibiotic resistance, especially against macrolides. Early studies, published in the late 90’s were conducted when clarithromycin had been approved for use for less than a decade and resistance was uncommon (Gardner et al., 2005), whereas resistance was reported as an issue by 2003, and this may have contributed to the reduced conversion rates recorded (Kobashi and Matsushima, 2003).

In 2020 an updated report on the guidelines for NTM treatment was published by the ATS, European Respiratory Society (ERS), *European Society of Clinical Microbiology and Infectious Diseases (ESCMID) and the* Infectious Disease Society of America (IDSA). The three-drug regime of ethambutol, rifampicin and a macrolide for treatment of *M. avium* was again endorsed, with the addition of amikacin liposome inhalation suspension (ALIS), approved by the FDA in 2018, for serious infection (U.S. Food and Drug Administration, 2018). If amikacin or streptomycin do not induce negative conversion within 6 months the less toxic ALIS is recommended. While new clinical evidence was used to inform additions to treatment, the report acknowledges the need for ‘well designed’ clinical trials to justify further alterations to this current, best available, treatment regime.

This same report, also defined the treatment regime for MAB. While it acknowledges that the optimal drugs, regimes and duration of treatment for *M. abscessus* is not known, a combination of at least three drugs including a macrolide, amikacin, imipenem, cefoxitin and tigecycline was recommended (Daley et al., 2020). If the strain is macrolide susceptible at least three drugs should be used, in macrolide resistant strains, treatments should include at least four drugs (Daley et al., 2020). *M. abscessus* is particularly challenging to treat, with success rates ranging between 41 and 46% for all subspecies (Diel et al., 2017b; Kwak et al., 2019). To date, no antibiotic regime based on *in vitro* research has effectively produced long term sputum conversion for patients with *M. abscessus* (Griffith et al., 2007). For all NTMs developing new drugs, with shorter, less toxic, treatment regimens are a research priority.

## 3 Clinical trials

As indicated, additional clinical trials, to determine the optimum treatment regime for each NTM infection, are urgently required. Although *in vitro* and *in vivo* studies are essential to understand drug and bacterial interactions, translation of promising *in vitro* data into positive clinical correlations has often not been forthcoming (Griffith et al., 2007). Between 85 and 90% of drugs fail the clinical trial phase (Ledford, 2011; Sun et al., 2022). Of those which succeed, only half are approved for clinical use (Ledford, 2011). Unmanageable toxicity accounts for 30% failure, while lack of clinical efficacy is responsible for 40–50% of clinical trial failures (Sun et al., 2022). Factors that influence this lack of efficacy include, variability in metabolic pathways and drug metabolism, complexity and regulation between species and animal models which may not accurately reflect human disease (Pound et al., 2004; Brubaker and Lauffenburger, 2020). For mycobacterial infections most drug discovery research is focused on TB, with few drugs in the NTM development pipeline designed specifically for NTM disease (Wu et al., 2018; Dartois and Dick, 2022). Currently, drugs that show promise in TB are usually then tested on NTM diseases. A focus on progressing drugs that target NTMs to clinical trials is required to effectively address this.

**Figure 1:**
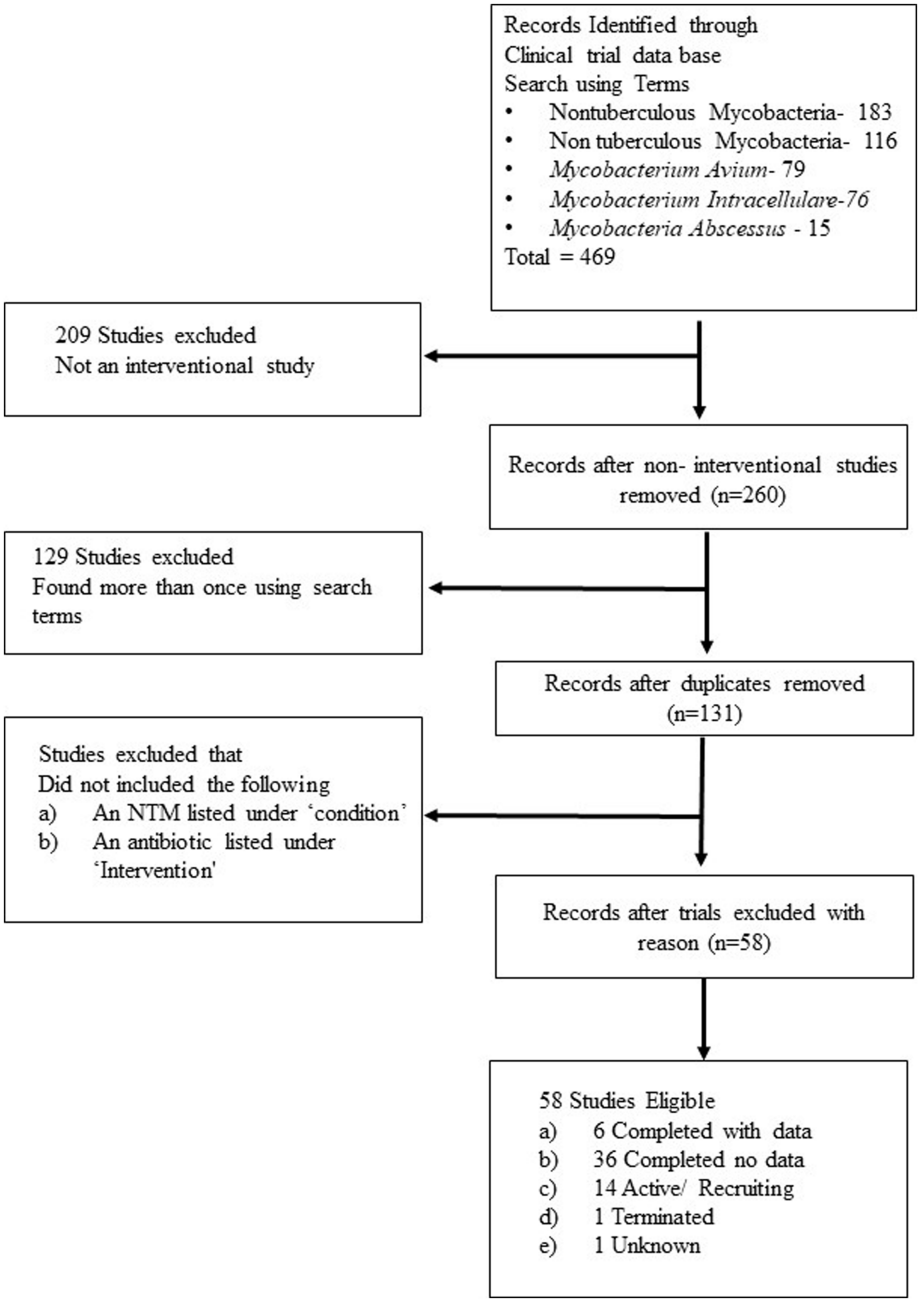
Flow diagram of clinical trial selection.

An assessment of registered clinical trials for NTMs on ClinicalTrials.Gov, and searching common NTM using the terms ‘Non tuberculous mycobacteria’, ‘Nontuberculous mycobacteria’, ‘*Mycobacterium. avium*’, ‘*Mycobacterium. intracellulare*’ and ‘*Mycobacterium. abscessus*’ and the selection criteria of clinical trial which (1) listed the use of an antibiotic, (2) was an interventional study and (3) listed an NTM under ‘condition’, identified 58 studies. Of these, 10 are listed as recruiting, 36 were completed with results to be released, and six provided results. One study was terminated, four were active not recruiting, with one with status unknown (Figure 1). The majority of these studies were testing a new antibiotic as monotherapy, or as an addition to the guideline-based therapy (GBT).

The trials with available data fall into two broad categories; studies looking at culture conversion following antibiotic use, or studies assessing pharmacokinetic drug interactions of antibiotics.

Of the trials assessing culture conversion, three utilized liposomal amikacin for inhibition (LAI), combined with standard therapy. The addition of LAI to a GBT regime significantly improved culture conversion, rising to 26.7–33.3% within 12 months compared to 9–13.7% for standard therapies (Olivier et al., 2017; Griffith et al., 2018; Winthrop et al., 2021). Although superiority of a LAI containing regime is shown it is notable that these conversion rates of GBT only are lower than the rates referenced above. The inclusion criteria for these studies were patients already receiving at least 6 months of GBT, within the last 12 month. Studies have shown that conversion within 6 months is predictive of treatment success, therefore it is not unexpected that these patients may have decreased conversion rates (Moon et al., 2019). Amikacin is an aminoglycoside primarily used for treatment of NTM infection when macrolide resistant forms are present. Side effects with amikacin are a significant issue, as amikacin treatment can cause irreversible ototoxicity and vestibular toxicity (Griffith et al., 2007). In the studies testing the addition of LAI (Table 2) it was administered as an inhalant. Adverse events, including COPD and bronchiectasis exacerbation, pneumonia, haemoptysis and worsening MAC infection remained high (Olivier et al., 2017; Griffith et al., 2021; Winthrop et al., 2021). LAI treatment was also successful in increasing conversion to culture negative for rapidly growing NTMs, with 50% conversion at 12 months, a 33% relapse rates and few serious adverse events (Siegel et al., 2023). An additional study adding the antibiotic tigecycline to GBT regimes for treatment of patients with *M. abscessus* and *M. chelonae* showed 60% improvement on culture conversion rates (Wallace et al., 2014).

**Table 2:** Completed clinical trials, assessing liposomal amikacin for inhalation.

NCT number	Study title	# Participants	Groups tested	Culture conversion rates	Adverse Events	Reference
NCT01315236	Liposomal Amikacin for Inhalation (LAI) for Nontuberculous Mycobacteria	90	LAI + GBT, Placebo +GBT	Within 84 days ALIS+GBT 32% GBT 9%	Adverse Events LAI + GBT 93.18% Placebo+ GBT 86.67% Serious Adverse Events LAI + GBT 18.18% Placebo+ GBT 8.89%	Olivier et al. (2017)
NCT02344004	Study to Evaluate Efficacy of LAI When Added to Multi-drug Regimen Compared to Multidrug Regimen Alone	336	LAI + GBT, GBT	Within 6 months ALIS+GBT 29% GBT 9%	Adverse Events LAI + GBT 89.24%, GBT 64.29% Serious Adverse Events LAI + GBT 20.18%, GBT 7.14%	Griffith et al. (2018)
NCT02628600	Open-label Safety Extension Study Assessing Safety and Tolerability of LAI in Patients Who Participated in Study INS-212	163	LAI + GBT Naïve, LAI + GBT prior exposure	Within 6 months ALIS Naïve 26.7% Prior ALIS cohort 9.6% Within 12 months ALIS Naïve 33.3% Prior ALIS cohort 13.7%	Adverse Events LAI + GBT naïve 83.33% LAI + GBT Prior 61.64% Serious Adverse Events LAI + GBT naïve 35.56% LAI + GBT Prior 27.4%	Winthrop et al. (2021)
NCT03038178	Liposomal Amikacin for Inhalation (LAI) in the Treatment of *Mycobacterium abscessus* Lung Disease	33	LAI+ combinations of Azithromycin, Clofazimine, Tigecycline, Imipenem and Linezolid	Within 12 months 50%		Siegel et al. (2023)
NCT00600600	Tigecycline for Treatment of Rapidly Growing Mycobacteria	8	Tigecycline	48.1% following treatment		Wallace et al. (2014)

LAI, liposomal amikacin for inhibition; GBT, guideline-based therapy.

Comorbidities and drug interactions with patients on treatment for other diseases such as *CF* and HIV pose additional challenges for treating NTM infections. The pharmacokinetic study, NCT01894776, revealed an antagonistic relationship between the antiretroviral drug, maraviroc and rifabutin. This antagonistic relationship, result in a suboptimal treatment responses for HIV+ patients (Abel et al., 2008). The relationship with treatments commonly prescribed for comorbidities such as *CF* and HIV in individuals being treated for NTM infection needs to be considered. This was recognized in 2015 by The US Cystic Fibrosis Foundation and the European Cystic Fibrosis Society in their joint statement that NTM disease poses a major threat to patients with *CF* (Floto et al., 2016). All current treatment guideline recommendations are based on data from patients without *CF* or extrapolated from studies with TB (Peloquin, 1997; Griffith et al., 2007). Additional studies for NTM infections to optimize treatment regimens need to consider the potential of antagonistic drug interactions.

### 3.1 Optimizing treatment regimes

Optimizing the current treatment regime and investigating new or modified antibiotic regimens is critical to increase cure rates and reduce relapse. The FORMat study (NCT04310930) (Table 3), is a multi-arm study ‘Finding the Optimal Regimen for *Mycobacterium abscessus* Treatment’. Other ongoing studies are introducing a new drug into the GBT, or studying the efficacy and safety of an isolated drug, which if successful these could lead to marked changes in therapy. The trial NCT05496374, is testing an aminobenzimidazole bacterial DNA gyrase -SPR720, which has demonstrated good activity against mycobacteria (Talley et al., 2021). If successful, SPR720 could be the first novel NTM antibiotic approved for use. The paucity of registered clinical trials for new NTM therapy is a major area of concern.

**Table 3:** Active NTM clinical trials.

NCT number	Study title	Mycobacteria	Treatment administered	Brief description of study	Phase	Estimated enrolment numbers
NCT03672630	Comparison of Two- Versus Three-antibiotic Therapy for Pulmonary *Mycobacterium avium* Complex Disease	MAC	Azithromycin, Ethambutol, rifampicin	To test whether a 2-drug antibiotic regime can be as effective as a 3-drug regime while maintaining efficacy and increasing tolerability	2&3	500
NCT04677569	Study to Evaluate ALIS (Amikacin Liposome Inhalation Suspension) in Participants With Nontuberculous Mycobacterial Lung Infection Caused by *Mycobacterium avium* Complex	MAC	Azithromycin, Ethambutol, ALIS, ELC (ALIS Placebo)	To evaluate efficacy of ALIS in combination with Azithromycin and Ethambutol compared to Azithromycin and Ethambutol with a placebo empty liposome container on patients respiratory symptoms	3	250
NCT02968212	Clofazimine in the Treatment of Pulmonary *Mycobacterium avium* Complex (MAC)	MAC	Clofazimine, placebo sugar pill	To evaluate the clinical efficacy and safety of clofazimine when used to treat MAC disease	2	102
NCT04287049	A Study of Standard Drugs for *Mycobacterium avium* Complex	MAC	Azithromycin	To assess the early bactericidal activity of Azithromycin over the first 14 days of treatment for MAC disease (monotherapy) followed by treatment with GBT	2	30
NCT03236987	CLArithromycin Versus AZIthromycin in the Treatment of *Mycobacterium avium* Complex (MAC) Lung Infections	MAC	Azithromycin, Clarithromycin, Ethambutol, Rifampicin	To compare efficacy of the macrolides clarithromycin and azithromycin when used in combination with ethambutol and rifampicin	3	424
NCT04630145	A Study of Bedaquiline Administered as Part of a Treatment Regimen with Clarithromycin and Ethambutol in Adult Patients with Treatment-refractory *Mycobacterium avium* Complex lung Disease (MAC-LD)	MAC	Bedaquiline, clarithromycin, ethambutol, rifampicin, rifabutin	To evaluate the efficacy of bedaquiline compared with rifamycin when administered with clarithromycin and ethambutol in participants with MAC disease for 24 weeks	2&3	124
NCT04310930	Finding the Optimal Regimen for *Mycobacterium abscessus* Treatment	MAB	Amikacin, Tigecycline, Imipenem, Cefoxitin, Azithromycin, Clarithromycin, Clofazimine, Ethambutol, Linezolid, Cotrimoxazole	Aims to produce high quality evidence for the best treatment regimens to maximize health outcomes and minimize toxicity and treatment burden to guide decisions for starting treatment and measuring disease severity in patients with MAB	2&3	300
NCT04921943	Hypertonic Saline for MAC	MAC	Hypertonic saline, Azithromycin, Ethambutol, Rifampicin	A study testing whether hypertonic saline helps improve symptoms and clearance of mycobacteria in patients with *M. avium* complex lung infections	4	50
NCT05861258	Pharmacokinetic Study of Minocycline in Patients with Pulmonary Nontuberculous Mycobacterial Disease (Mino-PK)	MAC	Minocycline	Pharmacokinetic study to assess exposure to minocycline in MAC-PD patients with and without concurrent use of rifampicin	2	15
NCT05496374	A Study to Evaluate the Efficacy, Safety, Tolerability, and Pharmacokinetics of SPR720 as Compared with Placebo for the Treatment of Participants with *Mycobacterium avium* Complex (MAC) Pulmonary Disease	NTM	SPR720 (500 mg and 1,000 mg)	To assess the safety, tolerability, pharmacokinetic and microbiological response of NTM patients to various doses of SPR720 compared to a placebo	2	31
NCT04922554	Oral Omadacycline vs. Placebo in Adults with NTM Pulmonary Disease Caused by *Mycobacterium abscessus* Complex (MABc)	MAB	Omadacycline Oral Tablet, Placebo tablet	To evaluate the efficacy, safety and tolerability of oral omadacycline as compared to placebo in adults with MAB	2	75
NCT05327803	Study of Epetraborole in Patients with Treatment-refractory MAC Lung Disease	MAC	Epetraborole + OBR, Placebo + OBR	To test the superiority of epetraborole + OBR compared to placebo + OBR in patients with MAC	2&3	314
NCT06004037	Study to Evaluate the Efficacy of Delpazolid as Add-on Therapy in Refractory *Mycobacterium abscessus* Complex	MAB	Delpazolid	To evaluate the efficacy and safety of delpazolid add-on therapy in Patients with Refractory *Mycobacterium abscessus* Complex Pulmonary disease	2	20
NCT04616924	RHB-204 for the Treatment of Pulmonary *Mycobacterium avium* Complex Disease	MAC	RHB-204 (Oral capsule containing a combination of clarithromycin, rifabutin, and clofazimine), placebo	To evaluate the efficacy and safety of RHB-204 in adult subjects with underlying nodular bronchiectasis and documented MAC lung infection.	3	125
NCT04921943	Hypertonic Saline for MAC	MAC	Hypertonic saline, Azithromycin, Ethambutol, Rifampicin	A study testing whether hypertonic saline helps improve symptoms and clearance of mycobacteria in patients with *M. avium* complex lung infections	4	50

MAC, *Mycobacterium avium* complex; MAB, *Mycobacterium abscessus*; ALIS, amikacin liposome inhalation suspension; ELC, empty liposome control; OBR, Optimized background regime.

## 4 Challenges to developing new therapies

### 4.1 Antibiotic resistance

Antibiotic resistance is one factor that makes effective treatment of NTM disease challenging. Resistance to NTMs can be either acquired or intrinsic. Unlike most resistance mechanisms which occur from horizontal transfer of mutations, mycobacterial resistance to antibiotics occurs most commonly through spontaneous mutations on chromosomal genes (Nasiri et al., 2017). Single point mutations on the 23 s rRNA gene (rrl) in positions 2058 or 2059 are the most commonly identified source of macrolide resistance (Meier et al., 1996; Wallace et al., 1996b). A mutation in the RNA polymerase beta subunit gene rPOB is responsible for 95% of rifampicin resistance and mutations on the emb CAB operon result in ethambutol resistance (Obata et al., 2006; Lingaraju et al., 2016).

Mutations can arise from a variety of different sources. NTM produce enzymes which can modify or neutralize antibiotics. This is achieved by preventing binding to the drug target or increasing susceptibility to hydrolysis by the bacteria, a classic example of this are beta lactamases (Luthra et al., 2018). Multiple NTM species also contain efflux pumps that contribute to decreased efficacy of antibiotics, by pumping antibiotics out of the cell into the external environment. Prolonged exposure of efflux pumps to NTM antibiotics, facilitated by long treatment times contribute to increased antibiotic resistance (Viveiros et al., 2003). Efflux pump inhibitors, such Verapamil can improve antibiotic success. Verapamil is a Ca^2+^ blocker, that decreases resistance to key NTM and TB drugs including rifampicin, isoniazid, streptomycin and bedaquiline (Song and Wu, 2016; Rodrigues et al., 2017). Finally, mutations in the transcription regulator whiB7, have a significant effect on susceptibility to mycobacterial infection, being associated with both hyper susceptibility and resistance (Warit et al., 2015; The Cryptic Consortium, 2022). Present in both pathogenic and non-pathogenic mycobacteria, whiB7 works on multiple pathways including antibiotic export and modifications of antibiotics and their targets (Burian et al., 2012; Cushman et al., 2021). WhiB7 transcription has been demonstrated to increase in the presence of antibiotics such as aminoglycosides and macrolides and confer various degrees of resistance (Burian et al., 2012). There have also been some reported instances where a frameshift mutation in whiB7 resulted in hyper susceptibility to clarithromycin (Warit et al., 2015). Using combinations of antibiotics lowers the instance of mutations, however, acquired resistance is not the only hurdle to effective treatment. Intrinsic resistance, such as the thick waxy cell wall and the proclivity to form biofilms also pose a significant treatment challenge.

Another challenge to effective treatment against NTMs is the difficulty in correlating *in vitro* drug susceptibility testing with clinical outcomes. Drug sensitivity testing for macrolides and amikacin demonstrate good clinical correlation, whereas minimum inhibitory concentration testing for rifampicin and ethambutol, do not show good correlation with clinical response (Daley, 2017).

### 4.2 Biofilms

In the environment *M. avium* often form biofilms, such as on PVC plastics in plumbing (Yamazaki et al., 2006b; Falkinham, 2011). Their slow growth rate, ability to adhere to other planktonic bacteria and to plastics, all facilitate their persistence in waterways (Falkinham, 2018a). Recent murine studies have also shown that *M. avium’s s*uccessful persistence *in vivo* is largely dependent on its ability to colonize and establish biofilms within the lung (Yamazaki et al., 2006a). Once established, it is incredibly difficult to kill bacteria in this form. The MBCs of *M. avium* in biofilms are four to six times higher than in planktonic form treated with a single antibiotic (Batista et al., 2023). As the majority of chronic and recurrent bacterial infections are caused by bacteria in biofilm, being able to penetrate, and break-down, the biofilm will be crucial to the success of new therapeutics (Mah, 2012).

### 4.3 Colony morphology

The smooth or rough appearance of NTM colony morphology can affect treatment. Although relevant in many species, colony morphology of *M. abscessus* is a key factor in treatment success. A retrospective multicentre cohort study conducted in Sweden between 2009 and 2020 found rough colony morphology was associated with worse treatment outcomes in patients with *M. abscessus.* Only 30% of patients achieved a clinical cure compared to 86% of patients with smooth morphology (Hedin et al., 2023). Mortality was also greater at 50% compared to 7% (Hedin et al., 2023). One key factor affecting colony morphology is the presence of glycopeptidolipids (GPL), with smooth colonies exhibiting higher GPL expression (Hedin et al., 2023). GPL also have a role in biofilm formation, with the smooth morphology *M. abscessus* forming biofilms by spreading out across the entire surface (Oschmann-Kadenbach et al., 2024). The exact mechanism for the poorer clinical outcomes with rough variants is unknown, however rough *M. abscessus* replicate more actively within macrophages and can spread more effectively between cells (Hedin et al., 2023). The rough variants also exhibit more cording, a virulence factor in mycobacterial infections known to inhibit macrophage phagocytosis (Howard et al., 2006; Hedin et al., 2023).

### 4.4 Cell wall

**Figure 2:**
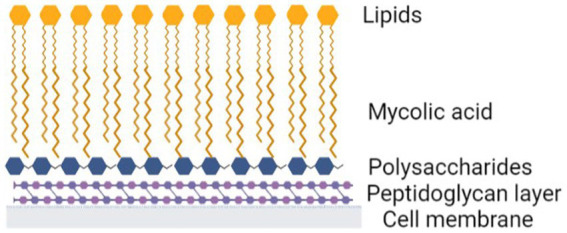
Mycobacteria cell wall. Created with Biorender.com

The unusual cell wall structure of mycobacteria also provides significant intrinsic resistance and a distinct survival advantage, enhancing viability and pathogenesis. Mycobacteria cell walls are highly lipid dense, lipids make up 60% of the cell wall mass (Kurz and Rivas-Santiago, 2020). This includes the outer lipids as well as mycolic acids. Mycolic acids are long chain acids comprising 60–90 carbon chains (Marrakchi et al., 2014; Minnikin and Brennan, 2020). Their structure contributes significantly to the low permeability of the mycobacterial cell wall (Liu et al., 1996). Mycolic acids attach to polysaccharides and then peptidoglycan and provide strength to the wall (Figure 2).

The cell wall is hydrophobic, while the majority of antibiotics are hydrophilic, and therefore cannot penetrate an intact cell wall (Falkinham, 2018b). Beta-lactams (β-lactams) and glycopeptides belong to a class of antibiotics which inhibit cell wall synthesis of both gram negative and positive bacteria (Batt et al., 2020). β-lactams, like penicillin and cephalosporins, work by irreversibly binding to the active sites of penicillin binding proteins and inhibiting 3 → 4 peptide crosslinking, hindering peptidoglycan synthesis of the cell wall (Kurz and Bonomo, 2012; Batt et al., 2020). Unfortunately, mycobacteria, have 3 → 3 peptide crosslinking, so are not inhibited by β-lactams(Kumar et al., 2012; Kurz and Bonomo, 2012). Glycopeptides, like vancomycin and teicoplanin also target peptidoglycan structures, inhibiting cell wall synthesis by binding to the C terminal acyl- D-ala-D-ala residues in peptidoglycan precursors (Reynolds, 1989). The thick waxy exterior of the NTMs hydrophobic outer layer, reduces the capacity of these glycopeptides to access the peptidoglycan layer thereby reducing the overall effectiveness of the antibiotic. Interestingly, mycobacteria have also developed additional strategies to survive cell wall disrupting antibiotics and can replicate in a cell wall deficient state.

## 5 NTMs and cell wall deficiency

When faced with antibiotic or nutrient stress NTMs are able to alter their morphology, forgoing their normal protective cell wall to grow in a cell wall deficient (CWD) state, previously called L-forms (Claessen and Errington, 2019). Without the cell wall, bacteria display as cocci (Mickiewicz et al., 2019). Cell wall deficiency enables NTMs to survive cell-wall targeting antibiotics and continue to replicate. CWD bacteria were first observed by Klieneberger in *Streptobacillus moniliformis* (Klieneberger, 1935). Initially speculated to be symbiosis between bacterial colonies, this theory was later abandoned by the author (Klieneberger, 1935; Klieneberger-Nobel, 1949). The discovery spurred on significant research into the phenomenon. Subsequent studies demonstrated that bacteria remain viable in these forms and revert back to their cell wall competent forms when the stress is removed (Mickiewicz et al., 2019). Studies in zebrafish have demonstrated that CWD can occur both *in vitro* and *in vivo* (Mickiewicz et al., 2019). The initial switch to a CWD form can occur rapidly in response to environmental factors. Multiple mycobacterial species, including *M. avium* and *M. tuberculosis*, can form viable CWD bacteria under chemical or cryogenic stress (Hines and Styer, 2003; Slavchev et al., 2016). It was initially thought that genetic alteration resulted in this morphological change, however, this was subsequently shown not to be the case, suggesting that changes in morphology were responsible for the increased antibiotic resistance (Slavchev et al., 2016). The potential to exploit the capacity of the bacteria to grow in a CWD state, as a treatment option is worth exploring. While in a CWD state the bacteria are resistant to cell wall inhibiting antibiotics, they may however, become more susceptible to other antibiotics, including those that are usually unable to penetrate the cell wall. Targeting synergy with new drugs combinations which induce CWD may provide alternatives to current treatment methods.

## 6 New options for NTM therapy

Treatment options for NTMs have largely been based on repurposing anti-TB antibiotics. This offers advantages in lower costs and less time to market. Increased instances of multi drug resistant (MDR) and extremely drug resistant (XDR) cases of TB have resulted in a greater focus on new pharmaceuticals with over 35 antibiotic candidates for TB treatment in discovery phase and approximately 30 in clinical trials (Wu et al., 2018). Exploring the options of repurposing antibiotics recently approved for TB, like bedaquiline and linezolid, use may identify new treatment options for NTM infections as well.

### 6.1 Bedaquiline

Bedaquiline was the first anti-TB drug approved for use by the FDA since rifampicin in 1971, approved against MDR and XDR-TB (World Health Organization, 2013; Nguyen et al., 2016). Bedaquiline acts by inhibiting ATP synthase and stopping mycobacterial F-ATP function, an affect specific to mycobacteria (Kundu et al., 2016). Low MICs have been shown when using this drug with good tolerability. The most significant adverse effect is a prolonging of QT intervals (Shulha et al., 2019) which needs to be monitored in patients. In TB studies, synergy has been shown between bedaquiline and other drugs including cephalosporins, linezolid and pyrazinamide (Khoshnood et al., 2021). While most data from this drug is on use against TB, one study has examined its effect on *M. avium*. This study found that the MIC90 was 0.015 μg/mL with 87% of isolates tested susceptible to bedaquiline at concentrations ≤0.008 μg/mL (Brown-Elliott et al., 2017), similar to the ranges those used for *M. tb* (Ismail et al., 2018). The inclusion of bedaquiline into XDR-TB treatment regimens lowered the risk of death compared to regimes not containing it (Schnippel et al., 2018). At least one listed clinical trial is currently recruiting to assess the effect of bedaquiline against NTM infection (Table 3).

### 6.2 Linezolid

Another antibiotic recently accepted for therapy against MDR and XRD TB is linezolid (Diekema and Jones, 2000). It acts by binding to 23 s and 50s ribosomal subunits of rRNA to inhibit bacterial protein synthesis at the initiation step (Diekema and Jones, 2000). The downside of linezolid is its high toxicity. A recent systematic review of 367 patients with MDR and XDR TB, found over 55% experienced some form of adverse effect (Zhang et al., 2015). Therapy was discontinued in 35% of these patients, most commonly occurring within the first 2 months (Zhang et al., 2015). Lower doses and shorter durations of antibiotics, by utilizing drugs with synergistic effects, could permit effective use of linezolid at reduced concentrations and thereby reduce toxicity. One study examining refractory NTM infection in 16 patients, demonstrated complete remission in 50% of patients and improvement in a further 25% but adverse reactions remained problematic (Chetchotisakd and Anunnatsiri, 2014). Newer drugs in this class of oxazolidinones are being developed for both TB and NTM infection, with the aim of having an improved safety and toxicity profile (Negatu et al., 2023). The outcome of ongoing studies to assess the effectiveness linezolid and bedaquiline to treat NTM infections are eagerly anticipated (Table 3).

### 6.3 Phage therapy

Bacteriophages, or phages, are viruses which infect and replicate exclusively within bacterial cells. They are highly diverse and found in abundance in the environment. Phages are host specific and often only infect a specific bacteria species or strain. Phages are absorbed by the bacterial host where they spread their genetic material, replicate and finally lyse the bacteria (Young et al., 2000). The use of phage therapy against NTM infection was first described in 2017 for two patients with *CF* and *M. abscessus* infection (Dedrick et al., 2019). The inclusion of phages, along with continued use of antibiotics, while not leading to complete clearance of the bacteria did generally result in improved clinical responses (Dedrick et al., 2019). A study across a small patient subpopulation receiving this therapy found phages successfully improved conditions in 75% of patients with a rough colony morphology (Dedrick et al., 2023). Several other studies using phages in humans also reported reducing NTM infection (Dedrick et al., 2019, 2021; Nick et al., 2022). Based on current research phages appear to be safe and well tolerated by patients. While resistance to bacteriophages has not been reported, host neutralization of phages may limit their effectiveness. There is also a considerable amount of personalization required for this therapy, making it more difficult to implement as a large-scale solution to NTM infection. Phages may be a useful tool, especially in combination with antibiotics. For large scale implementation, new antibiotics specific to NTM infections, are urgently required.

Expanding research to explore new antibiotics and therapeutic avenues that may demonstrate greater effectiveness against NTM disease is urgently required. Focusing on newly licensed anti-TB antibiotics, such as bedaquiline and linezolid in NTMs is one avenue, but new specific treatments will be required to manage the increasing public health threat posed by NTM disease.

## Author contributions

LC: Writing – original draft, Writing – review, editing. BS: Conceptualization, Funding acquisition, Supervision, Writing – original draft, Writing – review, editing.
